# Microenvironment in subchondral bone: predominant regulator for the treatment of osteoarthritis

**DOI:** 10.1136/annrheumdis-2020-218089

**Published:** 2020-11-06

**Authors:** Wenhui Hu, Yueqi Chen, Ce Dou, Shiwu Dong

**Affiliations:** 1 Department of Biomedical Materials Science, Third Military Medical University, Chongqing, China; 2 Department of Orthopedics, Southwest Hospital, Third Military Medical University, Chongqing, China; 3 State Key Laboratory of Trauma, Burns and Combined Injury, Third Military Medical University, Chongqing, China

**Keywords:** osteoarthritis, chondrocytes, cytokines, osteoarthritis, knee

## Abstract

Osteoarthritis (OA) is a degenerative joint disease in the elderly. Although OA has been considered as primarily a disease of the articular cartilage, the participation of subchondral bone in the pathogenesis of OA has attracted increasing attention. This review summarises the microstructural and histopathological changes in subchondral bone during OA progression that are due, at the cellular level, to changes in the interactions among osteocytes, osteoblasts, osteoclasts (OCs), endothelial cells and sensory neurons. Therefore, we focus on how pathological cellular interactions in the subchondral bone microenvironment promote subchondral bone destruction at different stages of OA progression. In addition, the limited amount of research on the communication between OCs in subchondral bone and chondrocytes (CCs) in articular cartilage during OA progression is reviewed. We propose the concept of ‘OC–CC crosstalk’ and describe the various pathways by which the two cell types might interact. Based on the ‘OC–CC crosstalk’, we elaborate potential therapeutic strategies for the treatment of OA, including restoring abnormal subchondral bone remodelling and blocking the bridge—subchondral type H vessels. Finally, the review summarises the current understanding of how the subchondral bone microenvironment is related to OA pain and describes potential interventions to reduce OA pain by targeting the subchondral bone microenvironment.

## Introduction

Osteoarthritis (OA) is the most frequent form of arthritis with a high incidence and a prolonged course.^1^ OA affects articular and periarticular tissues, such as articular cartilage, subchondral bone and synovium.^2^ Over recent years, the role of subchondral bone during OA progression has gradually attracted researchers’ attention.^3 4^ Imaging techniques have revealed microstructural alterations in subchondral bone in OA joints, including early-stage bone loss, late-stage bone sclerosis and histopathological alterations, caused by subchondral bone cysts, bone marrow oedema-like lesions (BMOLs) and osteophyte formation.^5^ These alterations are caused by biological processes involving uncoupling and coupling interactions among osteocytes, osteoblasts (OBs), osteoclasts (OCs), endothelial cells (ECs) and sensory neurons in the subchondral bone microenvironment,^6^ and therefore they will help in understanding OA pathogenesis from the perspective of subchondral bone. Notably, bone remodelling rates are altered during the development of OA due to the spontaneous activation or inactivation of osteoclastic bone resorption activity. As a result, activation of bone resorption may be evident in the subchondral bone microenvironment in early-stage OA, while late-stage OA is characterised by inactivation of bone resorption activity and a bias towards activation of bone formation activity.^7^ Subchondral bone and cartilage form a functional complex called the bone–cartilage unit, which is involved in the pathophysiology of OA at the biochemical and mechanical levels.^8 9^ In this review, we summarise the various pathways by which OCs interact with CCs, thus providing a novel research direction for the investigation of the crosstalk between these two types of cells in OA. Furthermore, we have noted the reported and potential communication pathways between OCs and CCs, and we propose promising therapeutic strategies to restrain the progression of OA by targeting the subchondral bone microenvironment. Moreover, arthritic pain is a major complaint of patients with OA during the progression of the disease. Recent studies indicate that neuronal factors may contribute to the innervation of pain-related sensory nerves in OA subchondral bone.^10 11^ Intriguingly, the evidence suggests a close relationship between OCs/OBs and sensory nerves in the microenvironment of subchondral bone.^10 11^ Based on this, it may be useful to develop specific drugs for the treatment of OA-related pain by targeting the subchondral bone microenvironment.

## Osteoarthritic subchondral bone microenvironment

### Normal subchondral bone architecture

Subchondral bone is divided into two anatomical entities: the subchondral bone plate and subchondral trabeculae. Subchondral bone plate is a thin cortical plate subjacent to calcified cartilage. It is a penetrable structure with interconnected porosity. Numerous vessels and nerves pass through the porosity, sending branches into calcified cartilage.^12^ The subchondral trabeculae, which are subjacent to the subchondral bone plate, are porous structures with abundant vessels and nerves that play an important role in load absorption and structural support as well as nutritional supply to cartilage.^13^ Subchondral bone adapts to mechanical forces exerted on the joint dynamically via coordinated bone remodelling.^14^ Bone remodelling involves the coupling of osteoclastic bone resorption and osteoblastic bone formation to replace damaged bone with new bone.^15^ However, subchondral bone and cartilage exhibit distinct capacities of mechanical adaptation. Although cartilage modulates the functional state in response to mechanical damage, its capacity to repair and modify the surrounding extracellular matrix is more limited than that of subchondral bone.^16^ Subchondral bone responds rapidly to mechanical loading by bone remodelling and then re-establishes normal physiological conditions.^17^

### Microstructural and histopathological alterations in OA subchondral bone

The occurrence of cartilage degeneration and subchondral bone destruction has always been a controversial issue.^18^ Not all patients with OA exhibit the progression from abnormal bone formation in subchondral bone. Moreover, a fraction of patients with OA exhibit the earliest changes at the sites of subchondral bone. OA is commonly thought to be a degenerative disease related to ageing and trauma. In ageing-induced OA, it could be confirmed that aberrant chondrocyte metabolism plays a crucial role in the occurrence of cartilage damage prior to abnormal subchondral bone formation.^19^ Conversely, early microdamage at the sites of subchondral bone is detected in trauma-induced OA.^20^ Notably, the alterations of subchondral bone are not exactly the same in different articulating joints in OA. There is good evidence that pathological alterations in different joints (such as the knee, spine and temporomandibular joint) exhibit several kinds of features.^21–25^

**Figure 1:**
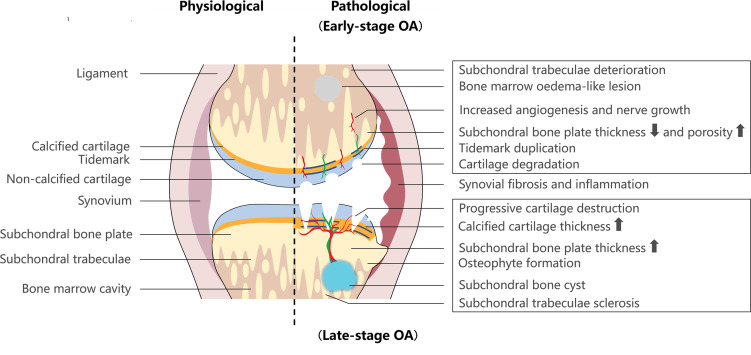
Microstructural and histopathological alterations in osteoarthritis (OA) subchondral bone. In early-stage OA, subchondral bone plate becomes thinner and more porous, together with deteriorated subchondral trabeculae and initial cartilage degradation. In late-stage OA, calcified cartilage and subchondral bone plate become thicker, along with sclerotic subchondral trabeculae and progressive cartilage destruction. During OA progression, growing vessels and nerves send branches from subchondral bone into cartilage. OA subchondral bone exhibits subchondral bone cysts, bone marrow oedema-like lesions and osteophyte formation.

At different stages of OA, there are distinct microstructural alterations in subchondral bone. In early OA, enhanced subchondral bone turnover is observed. In addition, the subchondral bone sclerosis is observed during the advanced and late stages.^26–28^ In early OA, subchondral bone plate becomes thinner and more porous during the initial cartilage degeneration. Subchondral trabeculae deteriorate, with increased trabecular separation and decreased trabecular thickness.^29^ Conversely, the subchondral bone plate and trabeculae become thicker, which is accompanied by subchondral bone sclerosis and decreased bone marrow spacing in late OA. At the same time, non-calcified cartilage shows progressive damage, and becomes thicker with tidemark replication.^29^ Despite the increased bone volume, high local bone turnover and a decreased calcium:collagen ratio lead to insufficient bone mineralisation and a decreased bone tissue elastic modulus. Consequently, the mechanical property is compromised, and it becomes easier to deform bone under mechanical loads (figure 1).^30 31^

### Abnormal cellular interactions in the OA subchondral bone microenvironment

Subchondral bone in OA undergoes an uncoupling of remodelling process, in which enhanced osteoclast-mediated bone resorption and osteoblast-mediated bone formation could be displayed at different stages during OA progression.^32^ Normally, biomechanical coupling of articular cartilage and subchondral bone has been well established. In early-stage OA, the self-repair of articular cartilage reduces excessive mechanical loads on subjacent subchondral bone. As a result, loading of subchondral bone falls below a predetermined level. In turn, this underloading increases the ratio of the expression of receptor activator of nuclear factor κB ligand (RANKL)/osteoprotegerin (OPG) in osteocytes, which leads to excessive osteoclastogenesis and enhanced bone resorption activity.^33 34^ Overactivated bone remodelling is commonly found at microdamage sites in subchondral bone in patients with OA and OA animal models.^35 36^ Osteocytes directly adjacent to microdamage sites undergo apoptosis, whereas osteocytes adjacent to apoptotic populations upregulate the expression of pro-osteoclastic molecules at the early stage of OA.^37 38^ Conversely, osteocytes also regulate osteoblast mineralisation by activating the Wnt signalling pathway via increased production of Wnt proteins and decreased secretion of sclerostin (SOST) in response to increased mechanical loading, which is caused by progressive cartilage destruction in OA during progression to the advanced and late stages.^39 40^ In addition, it was confirmed in vitro that transforming growth factor-β1 (TGF-β1) from osteocytes could enhance osteoblast-mediated bone anabolic metabolism by activating Smad2/3 in the subchondral bone in advanced-stage OA.^41^ As a result, the concomitant increase in osteoblast activity leads to spatial remineralisation and osteosclerosis in the end stage of OA.

In parallel, osteoclastic bone resorption leads to a sharp increase in active TGF-β1 in OA subchondral bone, recruiting osteoprogenitors to bone remodelling sites via activation of the Smad2/3 pathway to promote the formation of osteoid islets.^42^ Abnormal mechanical strain triggers dysregulated metabolism in osteoblasts, which is characterised by increased expression of interleukin (IL)−6, prostaglandin E2 (PGE2), the degradative metalloproteinases matrix metalloproteinase (MMP)–3, –9, −13 and RANKL and decreased production of OPG.^43^ IL-6 and PGE2 stimulate osteoclast formation by inhibiting the secretion of OPG and stimulating the production of RANKL in osteoblasts or by upregulating the expression of RANK in osteoclasts.^44^ Moreover, PGE2 promotes the secretion of IL-6, and in turn, IL-6 promotes the secretion of PGE2 by osteoblasts.^45^ Hence, the positive feedback loop between PGE2 and IL-6 signalling promotes osteoclast differentiation via affecting the OPG/RANKL/RANK system. In addition, RANKL and vascular endothelial growth factor (VEGF) secreted by osteoblasts in subchondral bone in OA could trigger osteoclast chemotaxis by inducing extracellular signal-regulated kinase 1/2 (ERK1/2) phosphorylation.^46–48^

**Figure 2:**
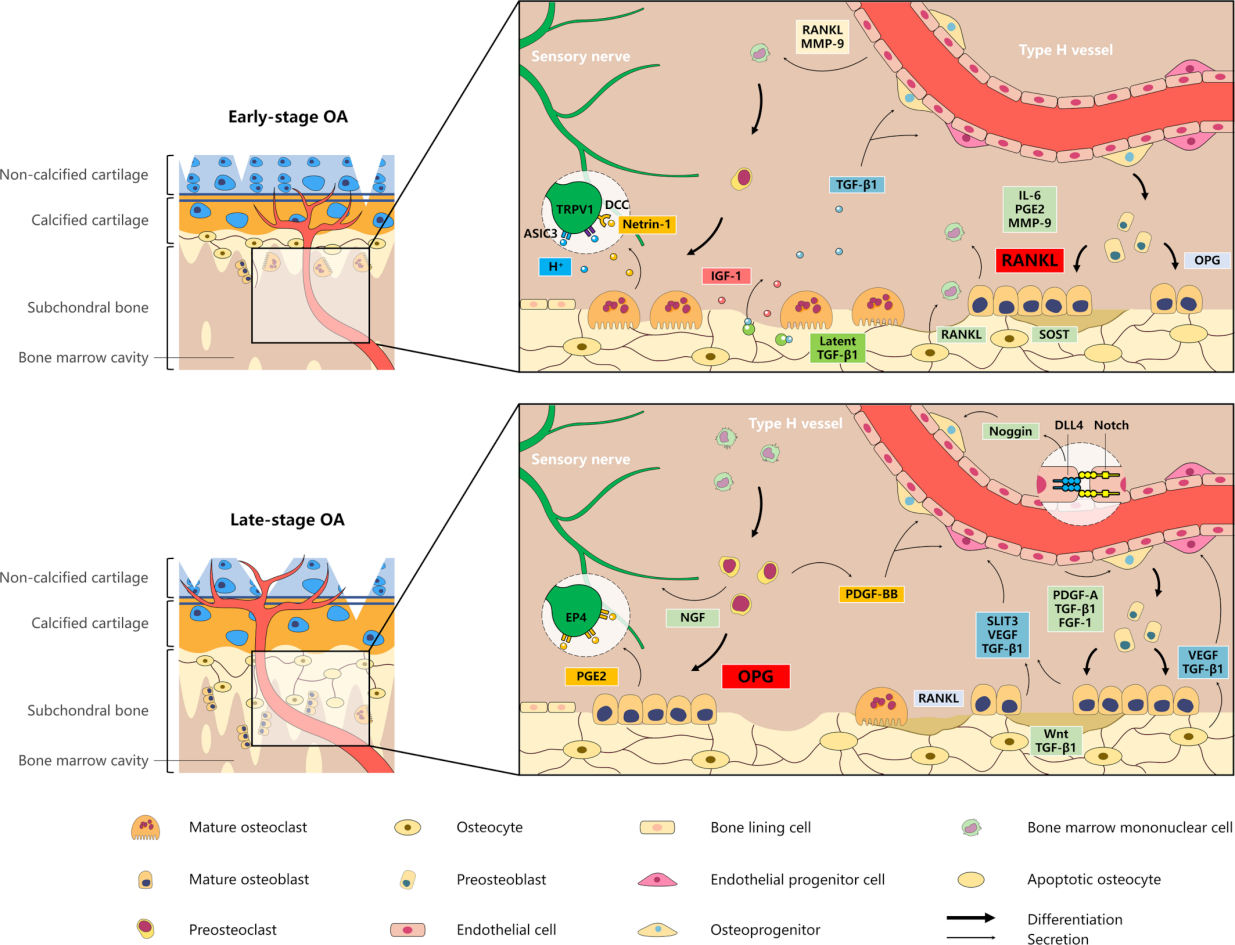
Pathological cellular interactions in the osteoarthritis (OA) subchondral microenvironment. (A) In early-stage OA, osteocytes upregulate the expression of RANKL:OPG ratio to enhance osteoclast differentiation. According to relative production of PGE2, IL-6 and OPG to RANKL, osteoblasts are separated into two subgroups: ‘low-synthesiser cells’ and ‘high-synthesisers’. PGE2, IL-6, MMP-9 and VEGF from the two subgroups mediate the pro-osteoclastic effect, while the former acts as primary effectors of subchondral bone loss by high levels of RANKL. In parallel, osteoclastic bone resorption is primarily responsible for angiogenesis and osteogenesis by released TGF-β1. Moreover, sensory innervation is induced by H^+^ and Netrin-1 secreted from mature osteoclasts. RANKL and MMP-9 produced by type H ECs may facilitate osteoclast chemotaxis and formation. (B) In late-stage OA, osteocytes regulate osteoblast mineralisation by increased Wnt proteins and TGF-β1 in response to increased mechanical loading. Multiple cells produce factors to support type H vessel formation, including VEGF and TGF-β1 from osteocyte, PDGF-BB from pre-osteoclasts, and VEGF, TGF-β1 and SLIT3 from osteoblasts. Sustained nerve sprouting is supported by NGF from preosteoclasts and PGE2 from osteoblasts. The latter subgroup promotes subchondral bone sclerosis, primarily regulated by angiocrine factors (PDGF-A, TGF-β1 and FGF-1). ASIC, acid-sensing ion channel; DCC, deleted in colon cancer; DLL4, delta-like protein 4; DP1R, DP1 receptor; IL-6, interleukin-6; MMP-9, matrix metalloproteinase-9; PDGF, platelet-derived growth factor; PG, prostaglandin; RANKL, receptor activator of NF-κB ligand; SLIT3, slit guidance ligand 3; SOST, sclerostin; TGF-β1, transforming growth factor-β1; TRPV1, transient receptor potential vanilloid 1; VEGF, vascular endothelial growth factor.

Evidence has shown that the crosstalk between osteoblast or osteoclast lineage cells and type H ECs promotes subchondral angiogenesis and aggravates subchondral bone remodelling.^49–51^ Type H ECs surrounded by osterix-expressing osteoprogenitors produce high levels of angiocrine factors (such as platelet-derived growth factor (PDGF)–A, TGF-β1 and fibroblast growth factor (FGF)−1), stimulating survival, proliferation and differentiation of these osteoprogenitors to promote local bone formation.^52 53^ Type H ECs intercommunicate via the intercellular Notch/delta-like protein 4 (DLL4) signalling pathway to induce the production of Noggin,^54^ which stimulates the differentiation of osteoprogenitors surrounding vessels.^55^ Type H vessels also stimulate osteoclast migration and differentiation by producing RANKL and MMP-9, which regulate bone remodelling to promote longitudinal bone growth.^56^ In addition, slit guidance ligand 3 (SLIT3) and TGF-β1 derived from osteoblasts acts as pro-angiogenic factors to increase the number of type H ECs.^57–59^ Notably, TGF-β1 derived from osteoclastic resorption is primarily responsible for subchondral angiogenesis in early-stage OA,^60^ while the increase in preosteoclast-derived PDGF-BB plays a relatively predominant role in angiogenic and osteogenic differentiation in late-stage OA (figure 2).^61^

## Regulation feedback loop of ‘osteoclast–chondrocyte crosstalk’

### Various pathways for the ‘osteoclast–chondrocyte crosstalk’

**Figure 3:**
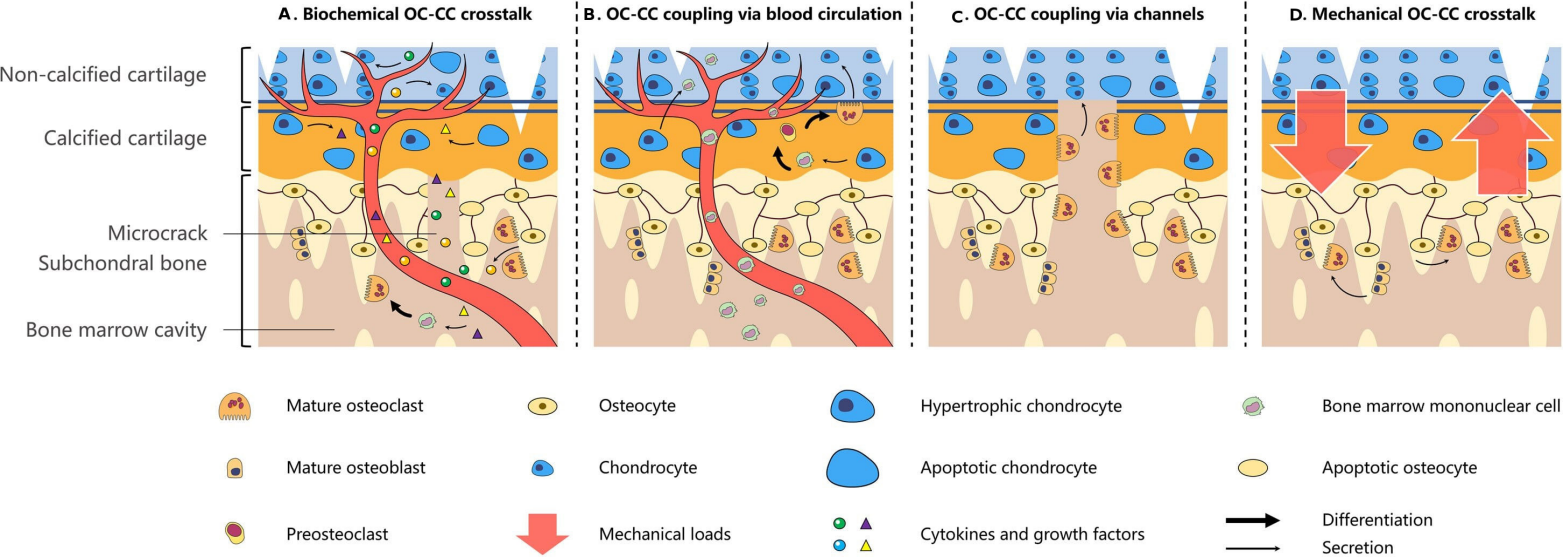
Various pathways for the ‘osteoclast–chondrocyte crosstalk’. (A) Osteoclasts (OC) and chondrocytes (CC) interplay through secreted mediators crossing microcracks and vessels. (B) Bone marrow mononuclear cells are brought to the cartilage layer through invasive vessels. Osteoclast lineage cells directly contact with chondrocytes at different stages of differentiation. (C) Mature osteoclasts tunnel their way into subchondral bone and overlying cartilage and interact with chondrocytes in the cartilage layer. (D) Subchondral bone destruction mediated by osteoclasts transfers shear forces to the cartilage layer and consequently leads to abnormal chondrocyte metabolism. In turn, osteocytes and osteoblasts sense overloads from the damaged cartilage layer and send pro-osteoclastic signals, resulting in accelerated subchondral bone remodelling.

A large number of vessels from subchondral bone penetrate calcified cartilage and invade non-calcified cartilage through vertical microcracks observed in OA joints.^62^ Consequently, mediators originating from osteoclasts and chondrocytes may diffuse and transport across microcracks or via invasive vessels. Intriguingly, osteoclast precursors invade the hypertrophic area of cartilage during the growth of periosteal vessels and then function together with hypertrophic chondrocytes to remodel cartilage matrix and form a primary ossification centre.^63 64^ Similarly, an in vivo cell tracking technique revealed that bone marrow–derived CX3CR1-positive osteoclast precursors enter the inflammatory cartilage layer via the blood circulation and differentiate into mature osteoclasts, promoting cartilage destruction in rheumatoid arthritis.^65^ Collectively, these data suggest that osteoclast precursors migrate into the cartilage layer and then make direct contact with hypertrophic chondrocytes and even interact with chondrocytes with normal phenotype. In addition, recent data have identified the capability of osteoclasts to degrade the osteochondral junction and articular cartilage in an MMP-dependent and cysteine protease–dependent manner,^66^ indicating the potential of mature osteoclasts to function as direct regulators of neighbour chondrocytes. During ‘mechanical OC-CC crosstalk’, on the one hand, the cartilage layer exhibits abnormal alterations in OA progression, which reduce its ability to absorb mechanical pressure and result in excessive loads on subchondral bone.^67^ On the other hand, high turnover of subchondral bone leads to alterations in the biomechanical properties of bone tissue in early OA, transferring shear forces to the cartilage layer and causing continued cartilage damage (figure 3).^68^

### Regulation of chondrocytes by osteoclasts promotes cartilage deterioration

Growth factors released from the bone matrix through osteoclastic bone resorption regulate chondrocyte metabolism and participate in cartilage deterioration. Mature osteoclasts attach to the bone surface through sealing zones and dissolve bone during bone remodelling. Consequently, various factors are released from the bone matrix, including TGF-β1, insulin-like growth factor (IGF)−1 and calcium-phosphate complexes.^69^ Zhang *et al* 70 found that the expression of TGF-β1 in osteoclasts was significantly upregulated in a time-dependent and dose-dependent manner under mechanical stimulation. Meanwhile, chondrocytes showed increased apoptosis when cultured with osteoclasts. Furthermore, intraperitoneal injection of TGF-β1R inhibitors reversed chondrocyte apoptosis and reduced cartilage degradation in a rat OA model.^70^ TGF-β1 is not derived from osteoclastic bone resorption in the study, no matter what, it implied that TGF-β1 in subchondral bone could be transferred to the cartilage layer by diffusion or blood transport to adversely affect chondrocytes. Intriguingly, IGF-1, another bone-released growth factor, was shown to play a protective role in chondrocyte anabolism. IGF-1 promotes the expression of Col2a1 and inhibits the expression and enzyme activity of MMP-13 by activating the phosphatidylinositol 3 kinase (PI3K)/Akt and ERK1/2 pathways in rat endplate chondrocytes.^71^ In addition, IGF-1 signalling protects chondrocytes from apoptosis by reducing caspase-3 activity and DNA fragmentation.^72 73^ Cartilage also obtains calcium–phosphate complexes from subchondral bone, which increases the production of MMP-13 in chondrocytes via activation of nuclear factor-kappa B (NF-κB), p38 and ERK1/2, and signal transducer and activator of transcription 3 (STAT3) signalling.^74^ Lu *et al* 50 reported a nutrient-sensing mechanism in which vascular-derived nutrients (such as amino acids) induced hypertrophic differentiation by activating mechanistic target of rapamycin complex 1 (mTORC1). Osteoclasts at distinct stages of differentiation derived from bone marrow mononuclear cells (BMMCs) may affect the normal phenotype of chondrocytes. Our group reported that exosomal let-7a-5p from preosteoclasts and mature osteoclasts targets Smad2 to promote the hypertrophic differentiation of chondrocytes,^75^ providing insights into ‘OC–CC coupling’ during OA progression.

### Regulation of osteoclasts by chondrocytes promotes subchondral bone loss

Subchondral bone cells may be exposed to various pro-inflammatory cytokines produced by OA chondrocytes. Changes in joint biomechanical properties induce the upregulation of IL-1β in primary chondrocytes.^76^ IL-1β upregulates the expression of RANKL by osteoblasts to indirectly induce osteoclast formation and directly induces osteoclast precursors to form multinucleated osteoclasts.^77^ The excessive production of tumour necrosis factor (TNF)-α and IL-6 in chondrocytes in OA was detected in a surgical OA model of destabilisation of the medial meniscus.^78^ TNF-α directly induces osteoclast differentiation by activating NF-κB and c-Jun NH2-terminal protein kinase (JNK) in a RANKL-independent manner^79^ and indirectly induces osteoclastogenesis by stimulating osteoblasts to express RANKL.^80^ IL-6 induces CD14-positive peripheral blood mononuclear cells to form tartrate-resistant acid phosphatase (TRAP) and calcitonin receptor–positive osteoclasts in a RANKL-independent manner by activating the signal transduction factor gp130.^81^ In addition, VEGF-positive and RANKL-positive chondrocytes are increased in the hypertrophic layer by applying mechanical stress to the temporomandibular joint. In parallel, TRAP-positive osteoclasts increase in the mineralised layer subjacent to the hypertrophic layer.^82^ Furthermore, RANKL and VEGF induced osteoclast chemotaxis through the phosphorylation of ERK1/2 in a modified model of osteoclasts cultured in a Boyden chamber.^83^ High-mobility group box 1 (HMGB1) is expressed in and around OA chondrocytes in vivo.^84^ Taniguchi *et al* 85 analysed the bone development of *Hmgb1* ^−/−^ in hypertrophic chondrocytes in the growth plate of mice and found that the endochondral bone formation was disrupted due to the delayed invasion of osteoclast precursors into the primary ossification centre. In addition, senescent chondrocytes occur alongside hypertrophic chondrocytes, which produce catabolic enzymes, pro-inflammatory mediators and chemokines (collectively known as the senescence-associated secretory phenotype (SASP)),^86 87^ potentially modulating the behaviours of subchondral osteoclast lineage cells.

**Figure 4:**
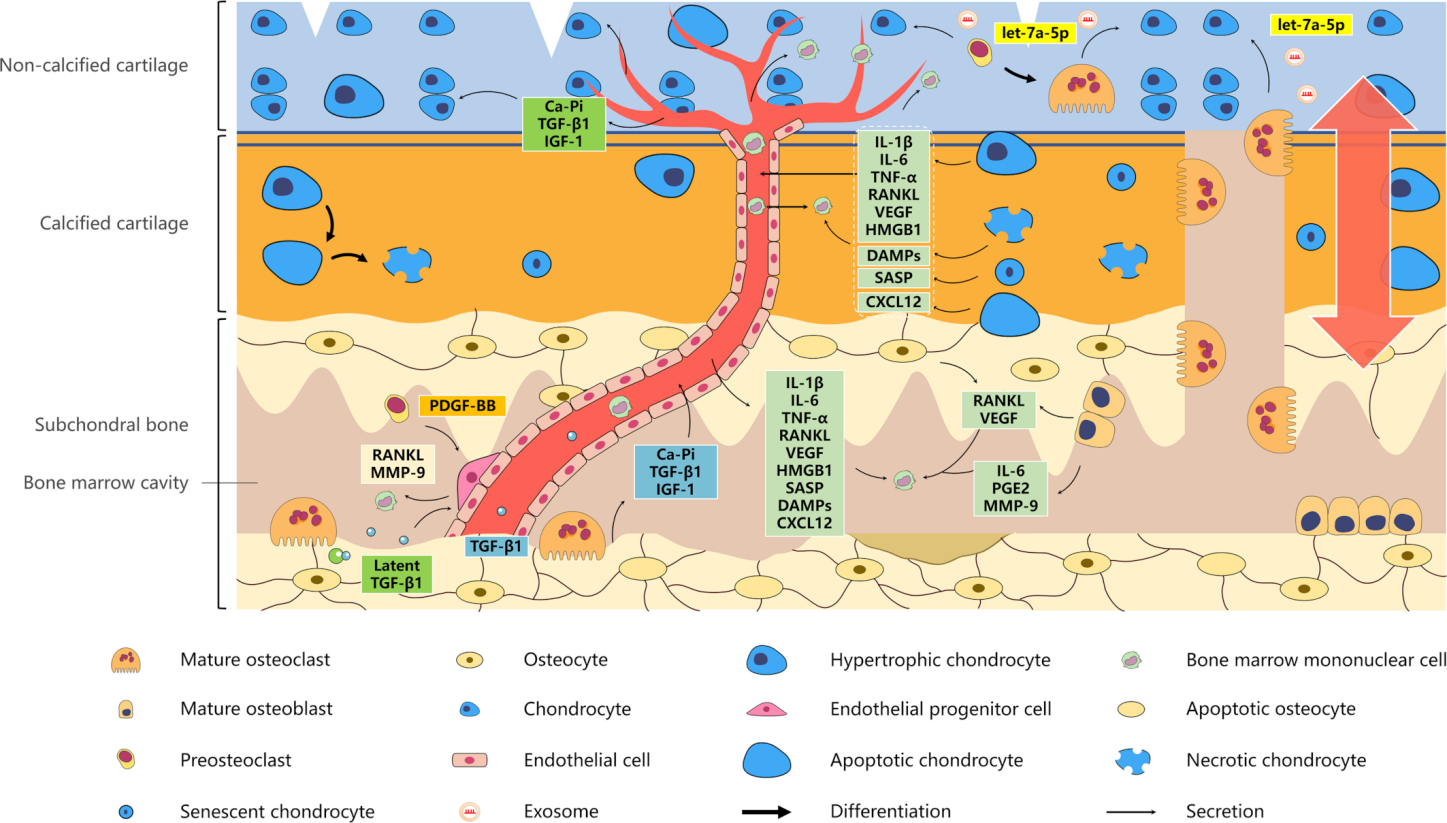
Role of the ‘osteoclast–chondrocyte crosstalk’ in the pathogenesis of OA. Multiple subchondral factors arrive at the cartilage layer through blood transport to regulate chondrocyte metabolism. For example, various factors are released from bone matrix, including TGF-β1, IGF-1 and Ca–Pi complexes. Moreover, BMMCs migrate into the cartilage layer. Mediators produced by chondrocytes are transported to the subchondral bone layer through blood transport. Hypertrophic, senescent and necrotic chondrocytes produce high levels of pro-osteoclastic molecules, which act on BMMCs in the subchondral bone or cartilage layer to promote osteoclast recruitment and formation. Preosteoclasts and mature osteoclasts in the cartilage layer induce chondrocyte hypertrophy through exosomal let-7a-5p. In addition, osteoclasts and chondrocytes influence each other by ‘OC–CC coupling via channels’ or ‘mechanical OC–CC crosstalk’. BMMC, bone marrow mononuclear cell; CXCL12, CXC motif chemokine 12; DAMP, damage-associated molecular pattern; FGF, fibroblast growth factor; HMGB1, high mobility group box 1; IGF, insulin-like growth factor; IL, interleukin; MMP-9, matrix metalloproteinase-9; PDGF, platelet-derived growth factor; PG, prostaglandin; RANKL, receptor activator of NF-κB ligand; SASP, senescence-associated secretory phenotype; SLIT3, slit guidance ligand 3; TGF-β1, transforming growth factor-β1; TNF-α, tumour necrosis factor-α; VEGF, vascular endothelial growth factor.

The presence of chondrocytes with morphological features consistent with apoptosis in OA cartilage is positively correlated with OA severity.^88–90^ Tang *et al* 91 found that the conditioned medium of apoptotic chondrocytes following dexamethasone treatment enhanced the recruitment of RAW264.7 osteoclast precursor cells and increased their differentiation potential. Further explorations confirmed that CXC motif chemokine 12 (CXCL12) released from apoptotic chondrocytes had the strongest pro-osteoclastic effect by activating the ERK1/2 and p38 pathways in BMMCs.^91^ AMD3100 (an inhibitor of CXCR4) effectively prevented subchondral trabecular destruction and cartilage loss in the tibia of mice after anterior cruciate ligament transection (ACLT).^92 93^ The cartilage matrix is the main obstacle for phagocytic cells, resulting in late apoptotic chondrocytes undergoing the transition to necrosis, which is called secondary necrosis.^94^ Necrosis causes plasma membrane rupture and the release of damage-associated molecular patterns (DAMPs), such as nucleotides, HMGB1 and pro-inflammatory cytokines.^95^ DAMPs act on nearby cartilage and synovium to trigger inflammation, and may regulate the behaviours of subchondral osteoclast lineage cells (figure 4, table 1).

**Table 1:** Role of the ‘osteoclast–chondrocyte crosstalk’ in the pathogenesis of OA

Origins	Factors	Effects	References
Bone resorption	TGF-β1	Induce endothelial progenitor cell and osteoprogenitor migration and chondrocyte hypertrophy and apoptosis	42 60 70
	IGF-1	Induce chondrocyte anabolism and prevent chondrocyte maturation and apoptosis	71–73
	Ca-Pi	Induce chondrocyte catabolism	74
Preosteoclast	PDGF-BB	Modulate chondrocytes through abnormal angiogenesis	61
	Exosomal let-7a-5p	Promote the hypertrophic differentiation of chondrocytes by targeting Smad2	75
Mature osteoclast	Exosomal let-7a-5p	Promote the hypertrophic differentiation of chondrocytes by targeting Smad2	75
Type H endothelial cell	MMP-9, RANKL	Stimulate osteoclast migration to indirectly affect chondrocytes	56
Mature osteoblast	IL-6, PGE2	Enhance osteoclast formation to indirectly regulate chondrocytes	43–45
	VEGF	Stimulate angiogenesis and osteoclast recruitment to indirectly affect chondrocytes	46 47
	RANKL	Stimulate osteoclast recruitment and differentiation to indirectly regulate chondrocytes	46 47
	MMP-9	Promote osteoclast recruitment to indirectly affect chondrocytes	43
	SLIT3, TGF-β1	Induce subchondral angiogenesis to indirectly affect chondrocytes	57–59
Osteocyte	VEGF, TGF-β1	Stimulate angiogenesis to indirectly regulate chondrocytes	33 34 41
	RANKL	Induce osteoclast recruitment and differentiation to indirectly modulate chondrocytes	33 34
Hypertrophic chondrocyte	IL-1β, IL-6, TNF-α	Induce osteoclast differentiation directly or indirectly	76–81
	RANKL, VEGF	Induce osteoclast chemotaxis and differentiation	82 83
	HMGB1	Promote osteoclast recruitment to indirectly affect chondrocytes	84 85
Senescent chondrocyte	SASP	Promote osteoclast chemotaxis and differentiation	86 87
Apoptotic chondrocyte	CXCL12	Enhance osteoclast recruitment and differentiation	91–93
Necrotic chondrocyte	DAMPs	Promote osteoclast formation	94 95

## Targeting the subchondrol bone microenvironment for the treatment of OA

### Restoring abnormal subchondral bone remodelling

In fact, the efficacy of antiresorptive agents in OA treatment has been evaluated in clinical trials by restoring abnormal subchondral bone remodelling. Regrettably, there are currently few or no data on the beneficial effect of strategies targeting abnormal bone remodelling in patients with OA. Bisphosphonates approved for osteoporosis management belong to classical antiresorptive agents. Risedronate reduced biochemical markers of cartilage degradation but did not improve signs or symptoms or slow radiographic progression in a prospective 2-year trial involving 2483 patients with medial compartment knee OA at dosages of 5 mg/day, 15 mg/day, 35 mg/week or 50 mg/week.^96^Alendronate treatment improved the Western Ontario and McMaster University Osteoarthritis Index pain score, decreased biochemical markers and increased the BMD in a prospective 2-year trial involving 50 patients with symptomatic hip OA.^97^ Moreover, compared with those receiving placebo, patients with symptomatic knee OA who received intravenous zoledronic acid yearly did not show a significant reduction in cartilage volume loss, the size of BMOLs or the pain score over 24 months.^98^ There are other antiresorptive agents (such as OPG, cathepsin K (CTSK) inhibitors and strontium ranelate) that may exert protective effects on subchondral bone and cartilage in animal models and serve as disease-modifying OA drugs for clinical treatment of OA.^99–101^ Intriguingly, calcitonin, which is known for targeting subchondral bone remodelling, also leads to intracellular cAMP accumulation and then promotes chondrocyte anabolism by binding to its receptors on human OA chondrocytes.^102^ Two phase III trials have reported a beneficial effect of bioactive oral calcitonin on joint pain and biochemical indicators of bone and cartilage degradation in patients with OA.^103^ Enhanced osteoclast activity leads to the overactivation of TGF-β1 signalling in subchondral bone, and therefore subchondral TGF-β1 is a pharmacological target for OA. Implantation of alginic acid microbeads with TGF-β1 antibody into subchondral bone or deletion of *Tgfbr2* prevented the phosphorylation of Smad2/3 in osteoblastic precursor cells, thus reducing their subchondral localisation and improving bone parameters and cartilage structure in a mouse ACLT model.^60^ Accumulating evidence suggests that restoring subchondral bone remodelling could improve OA symptoms and the structure of bone and cartilage, but these agents require large clinical trials with plenty of subjects to verify their effects.

### Blocking the bridge—subchondral type H vessels

Invasive subchondral type H vessels serve as a bridge between subchondral bone and articular cartilage. Current treatments for OA focus on the inhibition of inflammation and subchondral bone remodelling, while therapeutic strategies targeting subchondral angiogenesis are limited. In fact, blocking type H vessel formation in animal models of OA has been shown to reduce cartilage destruction and subchondral bone loss.^104^ For example, bevacizumab (a VEGF blocking antibody) attenuated the formation of subchondral type H vessels in an OA model, thereby inhibiting chondrocyte hypertrophy and delaying OA progression.^50^ In addition to pharmacological VEGF inhibition, secretory factors derived from osteoclast or osteoblast lineage cells in the OA subchondral bone microenvironment, such as TGF-β1, PDGF-BB and SLIT3, promote subchondral angiogenesis. Therefore, antagonists of those molecules might be developed as potential agents for OA. For example, the small molecule compound halofuginone inhibits Smad2/3-dependent TGF-β1 signalling to restore the coupling of subchondral bone remodelling, alleviate type H vessel formation and attenuate cartilage degradation in the rodent ACLT joint.^105^

### Ameliorating OA-related pain by modulating subchondral bone microenvironment

The detailed mechanisms of OA contributing to pain remained unclear for decades until recent studies found that particular neuronal factors related to aberrant bone remodelling cause the innervation of sensory nerves in the subchondral bone of patients with OA.^106 107^ Bone-resorbing osteoclasts create an acidic microenvironment by secreting H^+^ to cause bone pain in animal models of bone metastasis. Mechanistically, acidosis induces the expression and activation of acid-sensing receptor transient receptor potential vanilloid 1 (TRPV1) in dorsal root ganglions (DRGs). TRPV1 activation promotes extracellular Ca^2+^ influx and then activates calmodulin-dependent protein kinase II (CaMKII) and transcription factor cAMP-responsive element-binding protein (CREB), leading to the transcriptional activation of the pain-related molecule calcitonin gene-related peptide (CGRP).^108 109^ Similarly, acid-sensing ion channel 3 (ASIC3) is upregulated in mono-iodoacetate-induced OA model and is associated with hyperalgesia caused by increased Ca^2+^ influx.^110^ Netrin-1 secreted by osteoclasts induces sensory innervation and pain in OA through its receptor deleted in colon cancer (DCC).^10^ Preosteoclasts produce nerve growth factor (NGF), serving as key drivers of subchondral nerve innervation during OA development.^61^ In addition, PGE2 is synthesised by osteoblasts in response to low bone density and contributes to skeletal allodynia in OA mice by upregulating the voltage-gated sodium channel Na_v_1.8 and increasing Na^+^ influx in subchondral nociceptive neurons.^11^

Pain medications recommended in the current guidelines for OA include non-steroidal anti-inflammatory drugs, paracetamol, opioids and corticosteroids administered via the oral, topical or intra­articular route. Several new pain treatments are currently moving forward in preclinical and clinical evaluation processes, potentially marking the beginning of a new era in the management of OA-related pain. Tanezumab (a human monoclonal antibody against NGF) is significantly superior to placebo in reducing pain and improving joint function with fewer adverse events based on a meta-analysis of 10 studies.^111 112^ Evidence suggests that a small molecule conjugate linking the TGF-βR inhibitor TLY-2109761 and alendronate substantially reduces excessive PGE2 production by osteoblasts and alleviates OA-induced pain in OA mice by restoring aberrant bone remodelling.^11^ In addition, nociceptive signals were blunted in subchondral sensory neurons in OA mice by the administration of a cyclooxygenase 2 (COX2) inhibitor, the Na_v_1.8 inhibitor A-803467 and an EP4 receptor antagonist.^11 113 114^ Furthermore, Ca^2+^ influx into the cytoplasm in sensory neuron was inhibited by the TRPV1 antagonist SB366791 and the ASIC3 antagonist APETx2 to reduce acid-induced pain in a murine model of bone cancer pain and a rat model of OA, respectively.^110 115^ Collectively, further exploration of how the subchondral bone microenvironment is related to OA pain may be an excellent approach to develop specific drugs useful for the treatment of OA (table 2).

**Table 2:** Targeting the subchondral bone microenvironment for the treatment of osteoarthritis (OA)

Therapeutic strategy	Agents	Effects	References
Restoring abnormal subchondral bone remodelling	Bisphosphonate, osteoprotegerin, cathepsin K inhibitor, strontium ranelate	Relieve pain, improve joint structure, and reduce bone and cartilage degradation markers	96–101
	Calcitonin	Prevent bone pathology development and promote chondrocyte anabolism	102 103
	TGF-β1 inhibitor	Reform subchondral bone remodelling and inhibit subchondral angiogenesis	60
Blocking the bridge—subchondral type H vessels	Bevacizumab	Attenuate subchondral angiogenesis	50
	Halofuginone	Restore coupled bone remodelling and alleviate type H vessel formation by inhibiting TGF-β1 signalling	105
Ameliorating OA-related pain by modulating the subchondral bone microenvironment	Tanezumab	Reduce pain and improve joint function by binding NGF specifically	111 112
	SB366791, APETx2	Improve acidic subchondral bone microenvironment and acid-induced pain by inhibiting TRPV1 and ASIC3, respectively	110 115
	COX2 inhibitor, Na_v_1.8 inhibitor, EP4 receptor inhibitor	Blunt nociceptive signals in subchondral sensory neurons	11 113 114

## Conclusion and perspective

The bone–cartilage unit composed of subchondral bone and cartilage plays a significant role in joint homeostasis and OA development. During OA progression, the two joint compartments of the functional unit experience abnormal alterations in tissue structure and cellular activity. Therefore, therapeutic strategies targeting one of the abnormal joint compartments could restrain the progression of the pathology of the whole joint. Furthermore, this strategy may be an effective disease-modifying method to block pathological interactions between the two joint compartments through pharmacological interventions. More extensive cellular and molecular studies of bone–cartilage interface crosstalk will help us to better understand the pathophysiology of OA and modify existing OA therapies. In particular, the microenvironment in subchondral bone serves as the predominant regulator of the development of OA. Therefore, future studies should focus on how pathological cellular interactions in the subchondral bone microenvironment promote subchondral bone destruction and OA pain and the development of novel drugs to treat OA by targeting the subchondral bone microenvironment.
